# The study of statistical methods for evaluating the comparability of routine chemistry analytes among 3 routine laboratory measurement systems in China

**DOI:** 10.1186/s40064-016-3423-7

**Published:** 2016-10-06

**Authors:** Kun Zhong, Wei Wang, Chuanbao Zhang, Falin He, Shuai Yuan, Zhiguo Wang

**Affiliations:** 1 National Center for Clinical Laboratories, Beijing Hospital, National Center of Gerontology, No. 1 Dahua Road, Dong Dan, Beijing, 100730 China; 2 Beijing Engineering Research Center of Laboratory Medicine, Beijing, China

**Keywords:** Routine chemistry analyte, Mutual recognition, Statistical method, Comparability

## Abstract

**Background:**

Clinical laboratory tests are important for clinicians to make diagnostic decisions, but discrepancies may directly lead to incorrect diagnosis. We would like to introduce some statistical methods to evaluate the comparability of chemistry analytes while comparing the performances of different measurement systems.

**Methods:**

We used a panel of 10 fresh-frozen single donation serum samples to assess assays for the measurement of glucose and other 13 analytes. Statistical methods used in this article include traditional statistical analysis, robust statistics, regression analysis and differences on medical decision levels (MDL). All the statistical analysis results would be evaluated. 20 Chinese tertiary hospitals accredited to ISO 15189 took part in this work. The commercial random access platforms included: Olympus (8 labs), Hitachi (6 labs) and Roche (6 labs). To compare the acceptable rates, Chi square test was used.

**Results:**

The statistical analysis results are as follows: (1) Coefficient of variations are between 2.8 and 3.9 %, with the slopes and intercepts of regression functions between 0.928 to 1.064 and −0.174 to 0.630, respectively. (2) The percentage of robust *z*-scores between −2 and 2 is bigger than 90 %. (3) The total percentages of differences on all the MDLs are: less than optimal was 31.7 % (19/60); less than desirable was 60.0 % (36/60); less than minimum was 65.0 % (39/60); more than minimum was 35.0 % (21/60). In this study, 2 laboratories (Nos. 8 and 16) were considered as poor performance by z-scores. 10 laboratories (Nos. 4, 5, 7, 8, 9, 10, 11, 14, 16 and 19) have unacceptable measurement errors on MDLs. 10 laboratories (Nos. 1, 2, 3, 6, 12, 13, 15, 17, 18, 20) can achieve mutual recognition of serum glucose testing results, including: 5 (5/8) Olympus, 2 (2/6) Hitachi and 3 (3/6) Roche. There was no significant difference among acceptable rates of the three measurements systems for the serum glucose assay.

**Conclusions:**

Traditional statistical analysis, robust statistics and robust z-score, fitting linear regression equations and calculating differences on different MDLs can be used on studying the comparability and mutual recognition of clinical chemistry analytes among hospitals or laboratories in China. The mutual recognition and interchangeability of results remains jeopardized even among tertiary hospitals in China. More works and efforts should be done for improvement of the current situation of interchangeability of results in clinical laboratories in China.

## Background

The results of clinical laboratory tests can reflect the health status of patients, which are critical diagnostic evidences for clinicians. Performing accurate and precise measurements that are comparable over time and location and across assays is essential for ensuring appropriate clinical and public health practice (Stepman et al. 2014). Mutual recognition in clinical laboratory field is an agreement by which two or more laboratories agree to recognize one another’s test results of the same patient in a relatively short period, it is an aim of the health system. Before implementing mutual recognitions of clinical test analytes in China, patients were subjected to the same measurements repeatedly in different hospitals in a short period. These redundant measurements were not only a waste of time and medical expense, but also stressed patients due to repeated blood or other human sample collections. If the test results of different laboratories could achieve mutual recognition, analytes would not need to be duplicated measured in a reasonable short period. Mutual recognition is a regional agreement by which two or more laboratories agree to recognize one another’s testing results. In this article, 20 Chinese tertiary hospitals attended this study, and 14 clinical chemistry analytes were included. These analytes are (in serum): Alanine aminotransferase, Aspartate aminotransferase, Alkaline phosphatase, Glutamyltransferase, Lactate dehydrogenase, Creatine kinase, Urea nitrogen, Creatinine, Uric acid, Glucose, Total protein, Albumin, Cholesterol and Triglycerides. The analyte of serum glucose was used as the example for the statistical methodology study. In this article, the statistical methods and parameters, which may be useful for inter-laboratory comparison in China, would be calculated and analyzed, include: traditional statistical analysis of raw data, robust statistics and robust z-score, fitting linear regression equations and differences on medical decision levels (MDL). The interchangeability of results of serum glucose would be evaluated as example.

## Methods

### Ethics statement

The study involved use of leftover patient samples which were all de-identified during the collection. It was also ensured that appropriate amount of serum was collected from each patient sample so that a certain volume was left for possible repetition of measurement. The use of patient samples in the present study has been reviewed and approved by the Ethics Committee of Beijing Hospital and Shanghai Zhongshan Hospital. The authors and the related laboratories staffs confirmed that all subjects had given their consent to participate in this study even if the samples were the leftover samples from outpatient department. Our study adhered to strict ethical guidelines as set out by committee on publication ethics (COPE, http://publicationethics.org/).

### Laboratories and samples

We performed this study with 10 fresh-frozen single donation serum samples obtained from Shanghai Zhongshan hospital. Serum was collected according to the CLSI protocol C37-A without filtration and with 2 U/mL human thrombin (Sigma-Aldrich) added to the serum to facilitate clotting at room temperature (Wayne 1999). The individual blood donations were tested and found negative for anti-HIV I/II, anti– hepatitis C virus, and hepatitis B surface antigen. Immediately after 2-mL portions of the sera were aliquoted into polypropylene vials, the sera were stored at −70 °C and kept under these storage conditions until shipment on dry ice to the participating laboratories. The samples were required to be kept frozen until analysis. The participants (20 tertiary hospitals) each received 1 aliquot of the 10 samples, which was sufficient for analysis of the 14 analytes twice. The manufacturers/test systems used by participants were: Olympus AU (the serial numbers of labs were 1, 2, 11, 12, 15, 16, 17 and 19, n = 8), Hitachi (3, 4, 5, 7, 8 and 18, n = 6), and Roche Cobas (6, 9, 10, 13, 14 and 20, n = 6). The homogeneity and stability of the samples were guaranteed by National Center for Clinical Laboratories (NCCL) which prepared the samples as fresh frozen blood and had been approved by China National Accreditation Service for Conformity Assessment (CNAS) for ISO 17043. To compare the acceptable rates among measurement systems, Chi square (χ^2^) test was used. A *p* < 0.05 was considered significant.

### Traditional statistics and data treatment

All numerical results were converted to SI units. After calculating the median, arithmetic mean, standard deviation (s), coefficient of variation (CV), minimum and maximum, test results exceeding the range of arithmetic mean ± 3 times of s were considered as outliers and eliminated.

### Robust statistics analysis

Robust statistics (International Standard Organization 2005) are statistics that emulate popular statistical methods, but they are not affected by outliers or other small departures from model assumptions. Robustness is a property of the estimation algorithm, not the estimates it produces; therefore it is not strictly correct to call the averages and s calculated by such an algorithm robust. In order to avoid the use of excessively cumbersome terminology, the robust average and robusts should be understood in ISO 13528 as “mean estimates of the population mean” or “mean estimates of the population standard deviation calculated using a robust algorithm”. The robust estimates average and s were derived from an iterative calculation by updating the values of average and s several times from the modified data until the process converged.

The algorithm of robust average and robust s of robust statistics could be concisely described as below (International Standard Organization 2005):

Denote the submitted results of one lot, sorted into increasing order, by:

$${\text{x}}_{1} ,{\text{ x}}_{2} , \ldots ,{\text{ x}}_{\text{i}} , \ldots ,{\text{x}}_{\text{p}}$$

Denote the robust average and robust s of these data by ${\text{x}}^{*}$ and ${\text{s}}^{*}$.

Calculate initial values for ${\text{x}}^{*}$ and ${\text{s}}^{*}$ as:

$${\text{x}}^{*} \, = {\text{median of x}}_{\text{i}}\,\,\left( {{\text{i}} = 1, \, 2, \, 3, \, 4 \ldots ,{\text{ p}}} \right)$$

$${\text{s}}^{*} = 1.483 \, \times {\text{ median of }}\left| {{\text{x}}_{\text{i}} - {\text{ x}}^{*} } \right| \, \left( {{\text{i}} = 1, \, 2, \, 3, \, 4 \ldots ,{\text{ p}}} \right)$$

Update the values of ${\text{x}}^{*}$ and ${\text{s}}^{*}$ as follows. Calculate:

$$\delta \, = \, 1.5 \, \times {\text{ s}}^{*}$$

For each x_i_ (i = 1, 2, 3, 4…, p), calculate:

$${\text{x}}_{\text{i}}^{ *} = \left\{ {\begin{array}{*{20}l} {{\text{x}}^{*} - \, \delta ,} & {{\text{if}}\quad \, x_{i} < x^{*} \, - \, \delta } \\ {{\text{x}}^{*} \, + \, \delta ,} & {{\text{if}}\quad x_{i} > x^{*} \, + \, \delta } \\ {{\text{x}}_{\text{i}} ,} & {\text{otherwise}} \\ \end{array} } \right.$$

Calculate the new value of ${\text{x}}^{*}$ and ${\text{s}}^{*}$ from:

$${\text{x}}^{*} \, = \sum {\text{x}}_{\text{i}}^{*} /{\text{p}}$$

$${\text{s}}^{*} = 1.134 \, \times \left[ {\sum {({\text{x}}_{\text{i}}^{*} - {\text{x}}^{*} )2/({\text{p}} - 1)} } \right]^{0.5}$$

where the summation is over i.

The robust estimates ${\text{x}}^{*}$ and ${\text{s}}^{*}$ may be derived by an iterative calculation, i.e. by updating the value of ${\text{x}}^{*}$ and ${\text{s}}^{*}$ several times using the modified date, until the process converges. Convergence may be assumed when there is no change from one iteration to the next in the third significant figure of the robust s and of the equivalent figure in the robust average.

### Creating regression equation

The median of the results from each sample lot was treated as the independent variable (X), while the arithmetic average of the two test results was treated as the dependent variable (Y). Each regression equation for each laboratory was based on the median and its test results, Y = ${\text{k}}^{*}$X + b; where k is the slope, and b is the intercept.

### The differences of each MDL

The differences were calculated according to the regression equation and the MDLs of serum glucose, namely 2.50, 6.67 and 10.00 mmol/L (Statland 1987). The MDLs treated as independent variable (X) were brought into the regression equation to calculate the dependent variable (Y): the differences (%) = (Y − MDL)/(MDL) × 100 %. Compared to the desirable, optimum and minimum allowable differences derived from biological variation data (Joana et al. 2014), the inter-laboratory test results comparability and the differences on MDLs would be evaluated comprehensively.

### Calculating robust Z-score for every laboratories

Z-score is the standardized measurement of laboratory bias, which is calculated using the assigned value and the standard deviation for proficiency assessment. In this article, robust z-score (International Standard Organization 2005) was derived from the robust averages and robust s, and the formula for the robust z-score in this article is thus z = (x − X)/σ = (x – ${\text{x}}^{*}$)/${\text{s}}^{*}$, where x is the test results, X is the averages of x, σ is the s, ${\text{x}}^{*}$ is the robust averages, and ${\text{s}}^{*}$ is the robust s. Two or more robust z-scores of these 10 lots of one laboratory above 2 or below −2, shall be considered as poor performance and cannot be recognized with others.

## Results

### Traditional statistics

Only two single outliers were determined in lot 10, and the rest of the results were all in the range of arithmetic mean ± 3 times of s, for details please see Table 1.

**Table 1:** The traditional statistics of glucose test results (mmol/l)

Lot	n	Arithmetic mean	Median	s	CV (%)	Maximum	Minimum
1	40	6.02	6.00	0.23	3.8	6.43	5.54
2	40	7.13	7.11	0.26	3.6	7.90	6.62
3	40	5.12	5.11	0.19	3.8	5.58	4.67
4	40	5.74	5.75	0.21	3.6	6.09	5.18
5	40	6.67	6.65	0.26	3.9	7.10	6.00
6	40	7.62	7.62	0.28	3.6	8.15	6.90
7	40	8.79	8.80	0.26	2.9	9.37	8.14
8	40	9.94	9.97	0.30	3.0	10.55	9.06
9	40	14.42	14.50	0.40	2.8	15.27	13.43
10	38	11.98	11.96	0.39	3.2	12.75^a^	11.09

^a^The maximum is not including the outliers

### The regression equations and differences of each MDL

**Fig. 1:**
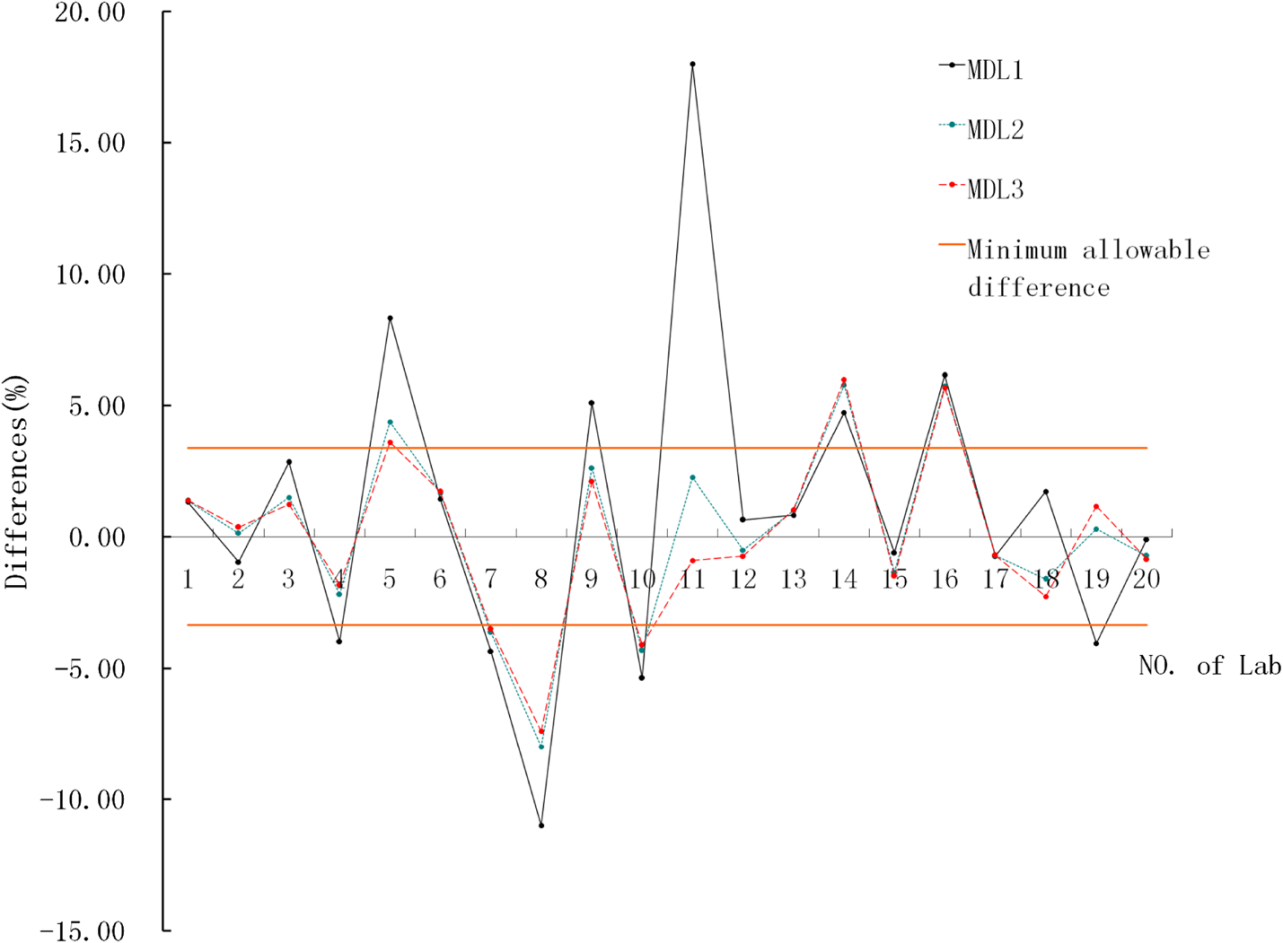
The differences of each medical decision level for all attended laboratories

After creating regression equations and substituting each MDL into the equations, the differences of each MDL for each of the 20 laboratories were calculated. The allowable differences of optimal, desirable and minimum of serum glucose were 1.17, 2.34 and 3.51 %, respectively, which were calculated from CVw (within-subject biologic variation) and CVg (between-subject biologic variation) of 2014 (Statland 1987). There were only 31.7 % (19/60) differences less than the optimal allowable bias, and 60.0 % (36/60) differences less than the desirable allowable differences and 65.0 % (39/60) for the minimum, suggesting there were more than 1/3 (21/60) differences that failed to meet the minimum allowable differences, see Table 2 and Fig. 1 for details. 10 laboratories (Nos. 4, 5, 7, 8, 9, 10, 11, 14, 16 and 19) have unacceptable measurement errors on MDLs.

**Table 2:** The regression equations and the differences in MDLs

No. of lab	Regression equation	Slope	Intercept	Correlation	Differences in MDLs (%)			Conclusion
				Coefficient	MDL1	MDL2	MDL3	
1	y = 1.014*x − 0.002	1.014	−0.002	0.9999	1.32	1.37	1.38	All less than desirable
2	y = 1.008*x − 0.044	1.008	−0.044	0.9997	−0.96	0.14	0.36	All less than optimal
3	y = 1.007*x + 0.053	1.007	0.053	0.9997	2.82	1.49	1.23	Two less than desirable
4	y = 0.989*x − 0.072	0.989	−0.072	0.9999	−3.98	−2.18	−1.82	Two less than desirable
5	y = 1.020*x + 0.158	1.020	0.158	0.9998	8.32	4.37	3.58	All bigger than min
6	y = 1.018*x − 0.009	1.018	−0.009	0.9994	1.44	1.67	1.71	All less than desirable
7	y = 0.968*x − 0.029	0.968	−0.029	0.9999	−4.36	−3.63	−3.49	One less than min
8	y = 0.938*x − 0.120	0.938	−0.120	0.9997	−11.00	−8.00	−7.40	All bigger than min
9	y = 1.011*x + 0.100	1.011	0.100	0.9994	5.10	2.60	2.10	Two less than min
10	y = 0.963*x − 0.042	0.963	−0.042	0.9999	−5.38	−4.33	−4.12	All bigger than min
11	y = 0.928*x + 0.630	0.928	0.630	0.9975	18.00	2.25	−0.90	Two less than desirable
12	y = 0.988*x + 0.046	0.988	0.046	0.9999	0.64	−0.51	−0.74	All less than optimal
13	y = 1.011*x − 0.007	1.011	−0.007	0.9999	0.82	1.00	1.03	All less than optimal
14	y = 1.064*x − 0.042	1.064	−0.042	0.9800	4.72	5.77	5.98	All bigger than min
15	y = 0.982*x + 0.030	0.982	0.030	0.9996	−0.60	−1.35	−1.50	All less than desirable
16	y = 1.055*x + 0.016	1.055	0.016	0.9998	6.14	5.74	5.66	All bigger than min
17	y = 0.993*x − 0.001	0.993	−0.001	0.9996	−0.74	−0.71	−0.71	All less than optimal
18	y = 0.964*x + 0.133	0.964	0.133	0.9999	1.72	−1.61	−2.27	All less than desirable
19	y = 1.029*x − 0.174	1.029	−0.174	0.9998	−4.06	0.29	1.16	Two less than optimal
20	y = 0.989*x + 0.025	0.989	0.025	0.9995	−0.10	−0.73	−0.85	All less than optimal

### Robust statistical results and robust Z-scores

Robust statistical results and the range of robust z-scores were listed in Table 3. The range of robust averages of all lots of samples were from 5.126 to 14.434 (mmol/l), robust standard deviations were from 0.179 to 0.433 with the change in the robust averages, and z-scores were from −2.895 to 5.356, except that lot 1 was only 80 % (16/20) of robust z-score in the range of −2 to 2 and others were no less than 90 % (18/20). The laboratories of Nos. 8 and 16 have two or more robust z-scores out of the range of −2 to 2.

**Table 3:** The robust statistics and robust z-scores^a^ of glucose test results

Lot	Robust average	Robust s	Range of robust z-scores	The percentage of |z-score| ≤2
1	6.009	0.179	−2.397 to 2.268	80 % (16/20)
2	7.117	0.209	−2.354 to 3.124	90 % (18/20)
3	5.126	0.186	−2.102 to 1.957	95 % (19/20)
4	5.746	0.193	−2.881 to 1.731	95 % (19/20)
5	6.683	0.264	−2.379 to 1.390	95 % (19/20)
6	7.630	0.254	−2.795 to 2.008	90 % (18/20)
7	8.795	0.209	−2.895 to 2.656	90 % (18/20)
8	9.949	0.270	−2.793 to 2.133	90 % (18/20)
9	14.434	0.377	−2.517 to 2.125	90 % (18/20)
10	12.026	0.433	−2.023 to 5.356	90 % (18/20)

^a^The z-score in this table have been derived from the data in column “Robust average” and “Robust s”. The formula for the z-score in this table is z = (x − ${\text{x}}^{*}$)/${\text{s}}^{*}$

### Laboratories/measurement systems performance

In this study, 10 laboratories (Nos. 4, 5, 7, 8, 9, 10, 11, 14, 16 and 19) have unacceptable measurement errors on MDLs of serum glucose. There were 3 (Nos. 11, 16 and 19) for Olympus AU, 4 (Nos. 4, 5, 7 and 8) for Hitachi and 3 (Nos. 9, 10 and 14) for Roche Cobas. There was no significant difference among acceptable rates of the three measurements systems for the serum glucose assay (data were not shown).

## Discussion

All hospitals and laboratories are eager to perform well. In order to achieve performing accurate and precise measurements, one method is to use assays that are metrologically traceable to a higher-order reference measurement system or harmonized by use of internationally recognized procedures (Vesper and Thienpont 2009; Miller et al. 2011). In Europe, the European Union Directive on in vitro diagnostic medical devices requires demonstration of metrological traceability (Directive 98/79/EC of the European Parliaments and of the Council of 27 October 1998). However, the intrinsic quality of a manufacturer’s assay or test system might be confounded by the laboratory using the system. German health reports showed that the unnecessarily repeated clinical laboratory tests cost 1.5 billion US dollars annually in 1998, while the United States cost 7.4 billion US dollars converted by the US GDP level. In 2004, NIST “The impact of calibration error in medical decision making (Final Report)” showed that only for one clinical test analyte—serum total calcium, the additional cost of calibration error was 0.06–0.199 billion US dollars (Michael). Duplicated clinical laboratory tests within a short period will result in the rising dissatisfaction of the patients to the healthcare providers and discrepancies in test results between different hospitals or from different instruments may directly lead to incorrect diagnosis or medical disputes.

The CV is a normalized measure of dispersion of a quantitative data. In this study the CVs were quite small which means the dispersion and differences among test results of all the laboratories were small. But currently there is no evaluation standard for evaluating the CVs of test results of more than one laboratory. The smaller CVs are, the better consistency of the test results are. The slopes (the closer to 1 the better) were used to evaluate the ratio errors of measurement system and intercepts (the closer to 0 the better) for evaluating the systematic errors. The performance of laboratory cannot be evaluated only by slope and intercept; the difference on the MDL which can be calculated by the regression equation is a useful indicator of performance statistics for laboratory. In this study, 10 laboratories (Nos. 4, 5, 7, 8, 9, 10, 11, 14, 16 and 19) have unacceptable measurement errors on MDLs. There were 3 labs for Olympus AU, 4 for Hitachi and 3 for Roche Cobas. There was no significant difference among acceptable rates of the three measurements systems for the serum glucose assay. Not same as the previous study in development country (Stepman et al. 2014). 10 laboratories (Nos. 1, 2, 3, 6, 12, 13, 15, 17, 18, 20) can achieve mutual recognition of serum glucose testing results and their differences on MDLs can be accepted.

In this study, arithmetic mean, median and robust average had little difference compared with each other in every lot of sample, which means that the dispersion of the results was small, and the distribution of the results was relatively concentrated.

When a participant reports a result that gives rise to z-score above 2 or below −2, then the result shall be considered to give a “warning signal”. In this study, two or more “warning signals” in one laboratory, shall be taken as evidence that an anomaly has occurred that requires investigation. In this study, 2 laboratories (Nos. 8 and 16) were considered as poor performance by z-scores.

To achieve the mutual recognition of clinical test results, the clinical laboratories should perform their daily work and management according to ISO 15189 as much as possible, internal quality control and external quality assessment should be performed timely, and the reference intervals should be the same with each other (Wang et al. 2011). On such basis, fresh clinical samples could be used to study the comparability of the results between the laboratories under the mutual recognition. In this article, we introduced the statistical methods and analyses including traditional statistics, robust statistics and robust z-scores, to create regression equations and calculate the differences of each laboratory in MDLs. The traditional and robust statistics described the basic information of the raw data, with the latter hardly affected by the outliers; robust z-scores described the locations of each test result in the overall data; the regression equations calculated the systemic errors of every laboratory in MDLs (the medical decision levels were substituted into the regression equation to evaluate the differences in MDLs). The methods described here were relatively intuitive and simple, easily applicable to office software or statistical software.

In this article we took the analyte of glucose as an example. The s and CVs (the test results differences between laboratories and the dispersion of the overall test results) were small in traditional and robust statistics. In total, more than 90 % of the robust z-scores were in the range of −2 to 2. They not only showed that the locations of every laboratory test results were within the overall results, but also indicated their differences from the robust averages. The three MDLs for glucose were 2.50, 6.67 and 10.00 mmol/l, and the allowable differences derived from biological variation data of optimal, desirable and minimum were 1.17, 2.34 and 3.51 %, respectively. In the MDLs for every attended clinical laboratory, there were only 31.7 % (19/60) of differences that met the optimal allowable difference, while 60.0 % (36/60) and 65.0 % (39/60) of differences that just couldmeet the desirable and minimum allowable differences, respectively. If one or more difference of a laboratory is more than minimum allowable difference, that shall be taken as poor performance that requires improvement. The smaller difference a laboratory had, the better comparability it would have and less difference could be found between this laboratory and overall ones. All the twenty laboratories demonstrated good linear and correlation with the entire concentration range: the slopes were close to 1 and the intercepts were close to 0, meanwhile the correlation coefficients tended to be 1. The statistical evaluation of robust z-scores was different from the differences evaluation of the regression equations because the statistical performance of robust statistics was not the same as traditional statistics.

In conclusion, all the statistical analysis methods mentioned in this article were designed for comparison and mutual recognition of clinical chemistry analytes. Calculating z-scores, fitting linear regression equations and calculating differences on different MDLs were used, and 10 laboratories were considered to achieve mutual recognition of the test results of serum glucose. All the statistical methods obtained good performances in the study of clinical chemistry analytes for comparison and mutual recognition among hospitals or laboratories using frozen fresh clinical samples. The mutual recognition and interchangeability of results remains jeopardized even among tertiary hospitals in China. More works and efforts should be done for improvement of the current situation of interchangeablity of results in clinical laboratories in China.
